# Self-rated health in Senegal: A comparison between urban and rural areas

**DOI:** 10.1371/journal.pone.0184416

**Published:** 2017-09-08

**Authors:** Priscilla Duboz, Gilles Boëtsch, Lamine Gueye, Enguerran Macia

**Affiliations:** 1 UMI 3189 ESS (CNRS/UCAD/UGB/USTTB/CNRST), Faculté de Médecine, de Pharmacie et d'Odontostomatologie, Université Cheikh Anta Diop de Dakar (UCAD), Dakar-Fann, Senegal; 2 Tessekere International Human-Environment Observatory (Labex DRIIHM, INEE, CNRS), Faculté de Médecine, de Pharmacie et d'Odontostomatologie, Université Cheikh Anta Diop de Dakar (UCAD), Dakar-Fann, Senegal; TNO, NETHERLANDS

## Abstract

**Introduction:**

Although the relationship between mortality and self-rated health has been demonstrated in sub-Saharan Africa, information in this area is rudimentary. In Senegal, no study has been undertaken comparing self-rated health between urban and rural areas. The objective of this study is therefore to compare self-rated health and its main predictors in Dakar and in a rural isolated area, Tessekere municipality, taking into account socio-demographic and economic factors, social relations, as well as measures of physical and mental health.

**Material and methods:**

This study was carried out in 2015 on a population sample of 1000 individuals living in Dakar and 500 individuals living in the municipality of Tessekere, constructed using the quota method. Self-rated health, health variables, psychosocial, sociodemographic and economic characteristics were collected during face-to-face interviews. Statistical analyses used were Chi-square tests and binary logistic regressions.

**Results:**

Results show that self-rated health in Senegalese urban area (Dakar) is better than in rural area (Tessekere), but the determinants of self-rated health partly differ between these two environments. Age and gender play a fundamental role in self-rated health as much in Dakar as in Tessekere but diabetes and social support play a role in self-rated health only in urban environment, whereas economic well-being is associated to self-rated health only in rural area.

**Conclusion:**

The analyses carried out in these two environments show that despite the existence of common determinants (age, gender, stress), the determinants for formulating an answer to the question of self-rated health differ. People’s social and cultural environments thus play a fundamental role in the process of rating one’s health and, in the short and long term, in the mortality rate.

## Introduction

Since the World Health Organization (WHO) gave a holistic definition of health in 1948 as “a state of complete physical, mental and social wellbeing and not merely the absence of disease, injury or infirmity” [1], the subjective aspect of health has captivated the attention of health researchers across disciplines. Heightened interest in subjective health is fuelled by increasing empirical evidence that how people feel about their own health is significantly related to numerous health outcomes (see [2] for a detailed review of the literature). Notwithstanding the acceptance of the WHO conceptualization of health around the globe, all efforts towards health improvement in sub-Saharan Africa tend to focus on objective health [3]. The neglect of the subjective aspect of health in the region is unfortunate, as studies have empirically established that subjective health is germane to the overall health status of a population [4,5].

### Epistemological and conceptual framework

Subjective health is a multi-dimensional notion that needs to be studied at the crossroads of disciplines [6]. From the anthropological perspective, the concept of subjective health can be conceptualized as encompassing biological, social and psychological dimensions of human beings [7]. One measure of subjective health that has garnered considerable attention in health studies is self-rated health (SRH). SRH is embodied by an individual’s instantaneous subjective evaluation of his or her own health status. It is assumed to capture numerous aspects of one’s health status. The general outlook on health that SRH seeks to capture is clearly articulated by Jyhlä: “Self-rated health, an individual and subjective conception that is related to the strongest biological indicator, death, constitutes a cross-road between the social world and psychological experiences on the one hand, and the biological world, on the other” [6].

There is a general contention in the literature that considering the subjective nature of SRH, people from different cultures are likely to evaluate their health differently [8,9]. The claim is basically that documented population differences in SRH may be attributable to cultural differences in the way of evaluating health, and even in defining the concept [6]. Notwithstanding the above issue(s), studies have found SRH to be a valid proxy for health across cultures [10]. Also, the World Health Survey and tests of the validity of SRH across cultures confirm that the instrument is as valid as any other measure of health status [11].

Studies have found SRH to have both constructs and criterion validity [12]. Research has documented that SRH has high reliability, validity, and predictive power for a variety of illnesses and conditions [13]. Above all, it has been found to be a valid measure of overall health status of a population [11]. A meta-analysis by Idler and Benjamini showed that in 23 of 27 studies, SRH reliably predicted survival in the population surveyed [4]. The predictive validity of SRH has been confirmed by some studies from developing countries as well. For example, based on longitudinal data from Indonesia, Frankenberg and Jones have reported that “individuals who perceive their health to be poor” were significantly more likely to die in subsequent follow-up periods than their counterparts who viewed their health as good, even after inclusion of measures of nutritional status, physical functioning, symptoms of poor physical health, depression, and hypertension [14]. Similarly, a study from Bangladesh has reported that self-reported health was significantly associated with measured physical performance among adult men and women aged 50 and older [15].

### Self-rated health in sub-Saharan Africa

In sub-Saharan Africa, information on self-rated health is rudimentary. The rare surveys conducted in this area have mainly focused on the social determinants of health, concentrating more on economic conditions and health inequalities [16–21], and even on social capital [22–24], than on objective health conditions. Moreover, most of this pioneering research focused mainly on specific age groups—older people, in particular [18,20,25–29]–making population-based generalizations impossible. For example, in Dakar, SRH was the subject of a specific study on a sample of adults aged 50 coming to a health-care centre [25]. This research showed that 81% of women in the sample gave a negative evaluation of their health, and that declared hypertension and age were associated with self-rated health. Of course, such results obtained from an aging population in particularly poor health cannot reflect SRH in the Senegalese general population.

The relationship between mortality and SRH has nevertheless been demonstrated in sub-Saharan Africa. In a recent study conducted by Ardington and Gasealahwe in South Africa, the association between reporting poor health and subsequent mortality is highly statistically significant for both men and women [30]. Furthermore, factors associated with SRH in this study are comparable to those identified in Western countries: men, people with a higher education level, married people and overweight people more commonly report they feel in good health than others. Still in South Africa, a study conducted on the social determinants of SRH have shown that elderly, single or divorced individuals as well as those with a lower education level also are more likely to report that they are in poor health [22]. Lastly, a recent study in Ouagadougou, the capital of neighboring Burkina-Faso, carried out on a general sample of adults (15 years old and over), took into account the physical and mental health of individuals [31]. Results showed that self-rated health is strongly associated with declared chronic illnesses and functional limitations in the Burkinabe capital, but that it did not correlate with depression. For the researchers, mental health is not a determinant of SRH in Ouagadougou—this being primarily understood as related to bodily functioning. Nevertheless, to better understand this major variable in health studies, cross-cultural comparisons are fundamental: determining variability and invariants are essential to any theory from an anthropological perspective.

### Rural and urban SRH: The case of Senegal

Several studies have been carried out comparing health indicators in urban and rural areas. Generally speaking, mortality is higher in rural areas than in urban areas in both developed countries [32–35] and in developing countries [36–38]. This can be explained by various favorable factors in cities, particularly socioeconomic and health factors. In urban areas, income is less irregular than in rural areas, and sanitation, preventive medicine and health care are all more available [39]. However, as regards SRH, studies comparing urban and rural areas are rarer: studies carried out in Korea [40], Ghana [41] and Finland [42] show that rural dwellers are more likely to report they are in ill health than urban dwellers, which is consistent with the lower mortality observed in the latter environment.

In Senegal, no study has been undertaken comparing SRH between urban and rural areas, whereas the influence of culture, including lifestyles and representations of illness and treatment, on the relationship between SRH and mortality has been shown on numerous occasions. It is therefore necessary to understand how the Senegalese rate their health and what the factors influencing this self-rating are, in order to improve public health measures and the health of the populations as well. The objective of this study is therefore to compare SRH and its main predictors in the Senegalese capital, Dakar, and in a rural isolated area, Tessekere municipality (Ferlo region), taking into account socio-demographic and economic factors, social relations, as well as measures of physical and mental health. From a scientific point of view, comparison of these results with those obtained in other cultures should also clarify variability and cross-cultural recurrence of the concept of subjective health.

To place this study in its particular context, clarification concerning Dakar and Tessekere municipality is necessary. First, we should point out that Senegal, according to United Nations, remains one of the least developed countries of the world [43]. As the political and economic capital of the country, the overall socio-economic situation appears much better in Dakar than in the rest of the country. In Dakar, for example, there is only 38% illiteracy (compared with 66.2% throughout the territory) and 94.4% of households have access to electricity, whereas Tessekere municipality is not equipped with electricity [44]. In addition, the people of Dakar spent 1,224 Francs CFA per day (i.e. 2 US Dollars) on average in 2005; or nearly three times more than in rural areas [45]. Regarding health, there are eight hospitals in the capital, and only three health care centers run by nurses in Tessekere.

Considering the gaps in the literature on subjective health in sub-Saharan Africa, the purpose of this paper is twofold. It is first to compare SRH in Dakar and Tessekere. Second, it sets out to elucidate the relative importance of the selected variables in predicting SRH in the two environments. Thus this paper is set to contribute to the literature concerned with SRH in sub-Saharan Africa using data from Senegal. Findings from this study are likely to provide an overview of the utility of SRH in a country where the epidemiologic and health transitions are underway.

## Material and methods

### Population sample

This study was conducted from November 2014 to June 2015 on a sample of 1,500 individuals aged 20 and older. The sample was constructed using the quota method (cross-section by age, gender and town of residence) in order to strive for representativeness of the population aged 20 and over living in the department of Dakar and Tessekere. For Dakar, a sample of 1,000 individuals was constructed on the basis of data from the National Agency of Statistics and Demography from the last census (2013). The quota variables used were gender (male / female), age (20–29 / 30–39 / 40–49 / 50–59 / 60–69 / 70 and over with an upper age limit of 100 years) and town of residence. The towns were grouped by the four *arrondissements* making up the department of Dakar: Plateau-Gorée (5 towns), Grand Dakar (6 towns), Parcelles Assainies (4 towns) and Almadies (4 towns). For Tessekere municipality, a sample of 500 individuals was constructed using the same population data, but as the area is less geographically extensive, the quota variables were solely gender and age.

Practically, the quota method requires constructing a sample that reflects the proportions observed in the general population. For example, according to the last census, men aged 20–29 living in the town of Medina (*arrondissement* of Plateau-Gorée) represented two per cent of the population aged 20 and over living in the department of Dakar. The sample was constructed to reflect this proportion and it included 12 men aged 20–29 living in this town. For each town, four doctoral-level investigators started out from different points each day to measure and interview individuals in Wolof or French in every third home. Investigators had a given number of individuals to interview (women aged 20–29 / men aged 20–29 / women aged 30–39 / men aged 30–39 etc., in each town) to meet the quotas. Only one person was selected as a respondent in each home. Face-to-face guided interviews based on a questionnaire were used to collect the data required for the study. The questionnaire contained items about socioeconomic characteristics, health and health-related quality of life, life satisfaction, economic well-being, as well as the quality of social support. Only a portion of the data collected was used for this article. Ethic approval was provided by the Comité National d’Ethique pour la Recherche en Santé (Protocole SEN 13/67).

### Dependent variable: Self-rated health

SRH was measured using a questionnaire with five possible answers: “Overall, would you say that your health is: excellent, very good, good, fair or poor?” For the majority of bivariate analyses and multivariate analyses, this variable was dichotomized. In accordance with Jylhä’s reflection [6] showing a break between good health–“the baseline that does not normally need to have a cause”–and less than good health, the split was made between the answers “excellent,” “very good,” and “good” (scored 0) and the answers “fair” and “poor” (scored 1).

### Health and psychosocial variables

*Blood pressure (BP)*: we used an OMRON M5-I digital automatic blood pressure monitor (OMRON^®^, s’Hertogenbosch, Netherlands) to take the participants’ blood pressure. Measurements were made on the upper right arm using an appropriate sized cuff while the participant was sitting and had rested for five minutes. Three readings were taken during the interview. The first was discarded, and the mean of the last two readings were used in the analysis. The first measurement was taken on both arms to detect a difference in blood pressure between arms. Hypertension was defined as a systolic BP ≥ 140 mmHg and/or a diastolic BP ≥ 90 mmHg or reported treatment for hypertension. Awareness of hypertension was defined as any self-reported prior diagnosis of hypertension by a health care professional among the population defined as having hypertension. Hypertensive aware participants were classified as being on treatment if they reported current use of drugs prescribed by a health professional which they had taken within the past two weeks prior to the study. Control was defined as the proportion of the sample on antihypertensive therapy with BP <140/90mmHg.

*Body Mass Index*: Following WHO recommendations, BMI was calculated by dividing weight (kg) by the square of the height (m^2^). Weight was measured using a digital scale (measurement accuracy of 100 g), with subjects dressed in minimum clothing and barefoot. To measure height, the subject was to stand “to attention,” arms at sides, heels joined. Thinness was defined as BMI < 18.5; normal weight as 18.5 ≤ BMI < 25; overweight as 25 ≤ BMI < 30; whereas obesity corresponded to a BMI of ≥ 30. For the sake of analyses, people with excess weight (BMI ≥ 25) were distinguished from others (BMI < 25).

*Diabetes*: subjects were examined during the morning after fasting since the previous evening meal. The day before the investigation, subjects were informed of the need to have nothing to drink or eat in order to measure capillary whole blood glucose. Capillary whole blood (glucose) was obtained from a finger prick and was immediately analyzed using a Hemocue blood glucose analyzer^®^. Participants were then divided into two categories according to international standards: those without diabetes, for whom fasting plasma glucose levels < 125 mg/dL; and those with diabetes, who had either been previously diagnosed diabetics or had capillary whole blood glucose value greater than or equal to 126 mg/dL.

*Stress*: Perceived Stress Scale [46] was used to measure psychosocial stress in individuals. Six out of the ten items of PSS-10 are considered negative (1, 2, 3, 6, 9, 10) and the remaining four as positive (4, 5, 7, 8), representing perceived helplessness and self-efficacy, respectively. Each item was rated on a five-point Likert-type scale (0 = never to 4 = very often). Total scores are calculated after reversing positive items’ scores and then summing up all scores. Total scores for PSS-10 range from 0 to 40. A higher score indicates greater stress.

*Social support*: Social support was measured by asking respondents “If you were in trouble, do you have friends and relatives you can count on to help you whenever you need them, or not?” [47]. Answers were coded 1 when the answer to the question was affirmative, and 0 when negative.

### Socio-demographic and economic variables

#### Economic conditions

The following question was used as an indicator of economic conditions: “Given your household income, do you feel you … a) live well? b) live okay? c) live okay, but you have to be careful? d) have difficulty making ends meet?” This question, taken directly from Razafindrakoto and Roubaud’s study, has demonstrated validity and relevance in eight African capitals, including Dakar, to measure economic conditions in the context of subjective well-being [48]. For the analyses, the answers were coded from 1 (poor) to 4 (prosperous).

#### Socio-demographic variables

Among the socio-demographic data collected during the interviews, four were taken into account for this study: age (20-29/30-39/40-49/50 and over), gender (male/female), educational level—defined in accordance with the educational system in Senegal—(0/1-5/6-9/10-12/over 12 years of school) and marital status (single/divorced/married/widowed).

### Statistical analyses

Bivariate and multivariate analyses were used to test associations between dependent and independent variables. Bivariate analyses included Chi^2^ tests, and student’s t test for mean comparisons. The Chi^2^ tests were performed to compare the distribution of good/poor self-rated health among groups defined by age, gender, educational level, marital status, economic conditions, social support, HTN, BMI and diabetes. Student’s t test was used to compare mean stress scores between self-rated health (1 vs 0). Logistic regression was then used to assess the extent to which the various factors assessed predicted poor self-rated health. The software used for the statistical analysis was IBM SPSS Statistics 22.

## Results

### Sample characteristics

Sample characteristics are presented in Table 1. The results show that the Dakar population sample is significantly younger and better educated than the Tessekere sample. The number of singles is also higher in Dakar, as is the number of people saying they live well on their household income. On the other hand, the number of people who say they can rely on social support is lower in Dakar than in Tessekere. As for the biological variables, significantly fewer Dakar residents appear to suffer from high blood pressure, but more of them are overweight or obese than Tessekere inhabitants. No difference was noted in the distribution by gender, glycemia or stress. Finally, the rural dwellers of Tessekere rate their health significantly more negatively than Dakar’s urban dwellers: 43% and 30.3% respectively rate their health negatively (Chi^2^ test result).

**Table 1:** Socio-demographic and economic characteristics of the general sample (N = 1500).

Variables	Categories	DAKAR		TESSEKERE		Total	Test
		N	%	N	%		
Sex	Men	494	49.40	241	48.20	735	χ^2^ 0.192; p 0.661
	Women	506	50.60	259	51.80	765	
Age brackets	20–29	424	42.40	203	40.60	627	χ^2^ 8.678; p 0.034
	30–39	269	26.90	116	23.20	385	
	40–49	157	15.70	77	15.40	234	
	≥ 50	150	15.00	104	20.80	254	
Marital status	Single	430	43.00	66	13.20	496	χ^2^ 159.820; p < 0.001
	Divorced	55	5.50	12	2.40	67	
	Married	472	47.20	402	80.40	874	
	Widowed	43	4.30	20	4.00	63	
Education level	0 year	209	20.90	376	75.20	585	χ^2^ 428.940; p < 0.001
	1–5 year(s)	359	35.90	87	17.40	446	
	6–9 years	199	19.90	18	3.60	217	
	9–12 years	92	9.20	13	2.60	105	
	> 12 years	141	14.10	6	1.20	147	
Social support	No	163	16.30	40	8.00	203	χ^2^ 19.624; p < 0.001
	Yes	837	83.70	460	92.00	1297	
Economic well-being	Have difficulty making ends meet	109	10.90	73	14.60	182	χ^2^ 57.650; p < 0.001
	Live ok but have to be careful	161	16.10	156	31.20	317	
	Live ok	559	55.90	212	42.40	771	
	Live well	171	17.10	59	11.80	230	
Glycemia	< 126 mg/dL	957	95.70	479	95.80	1436	χ^2^ 0.008; p 0.928
	≥ 126 mg/dL	43	4.30	21	4.20	64	
Body Mass Index	BMI < 18.5	124	12.40	149	29.80	273	χ^2^ 84.558; p < 0.001
	18.5 ≤ BMI < 25	582	58.20	270	54.00	852	
	25 ≤ BMI < 30	197	19.70	66	13.20	263	
	BMI ≥ 30	97	9.70	15	3.00	112	
Arterial hypertension	No hypertension	754	75.40	343	68.60	1097	χ^2^ 7.845; p 0.005
	Hypertension	246	24.60	157	31.40	403	
Stress	Mean	16.54 ± 5.684		16.96 ± 5.963			t 1.318; p 0.188
Self-rated health	Good	697	69.70	285	57.00	982	χ^2^ 23.781; p < 0.001
	Poor	303	30.30	215	43.00	518	
Total		1000	100.00	500	100.00	1500	

### Bivariate analyses by territory

In Dakar municipality, Chi^2^ tests performed revealed significant associations between self-rated health and all variables taken into consideration except education level: people aged 50 and over, women, people living with difficulty and those with HTN and diabetes rate their health significantly worse than, younger people, men, people living ok or well and people without chronic diseases respectively. Furthermore, singles, thin and normal-weight people, people with little stress and those who enjoy social support rate their health more positively (see Tables 2 and 3). In Tessekere, the results differ: education level is correlated with self-rated health (people with a year or more of education rated their health more positively), whereas diabetes, BMI and social support do not appear to be associated with self-rated health.

**Table 2:** Socio-demographic and psychosocial characteristics of the general sample by self-rated health (N = 1500).

DAKAR									TESSEKERE						
Variables	Categories	Very poor/Poor		Good/very good/Excellent		Total		Test	Very poor/Poor		Good/very good/Excellent		Total		Test
		N	%	N	%	N	%		N	%	N	%	N	%	
Sex	Men	117	38.61	377	54.09	494	49.40	Chi^2^ = 20.233; <0.001	87	40.47	154	54.04	241	48.20	Chi^2^ = 9.038; 0.003
	Women	186	61.39	320	45.91	506	50.60		128	59.53	131	45.96	259	51.80	
Age brakets	20–29	105	34.65	319	45.77	424	42.40	Chi^2^ = 30.397; <0.001	49	22.79	154	54.04	203	40.60	Chi^2^ = 66.442; <0.001
	30–39	69	22.77	200	28.69	269	26.90		51	23.72	65	22.81	116	23.20	
	40–49	61	20.13	96	13.77	157	15.70		41	19.07	36	12.63	77	15.40	
	≥ 50	68	22.44	82	11.76	150	15.00		74	34.42	30	10.53	104	20.80	
Education level	0	73	24.09	136	19.51	209	20.90	Chi^2^ = 6.217; 0.184	183	85.12	193	67.72	376	75.20	Chi^2^ = 21.068; <0.001
	1–5	112	36.96	247	35.44	359	35.90		25	11.63	62	21.75	87	17.40	
	6–9	61	20.13	138	19.80	199	19.90		4	1.86	14	4.91	18	3.60	
	9–12	24	7.92	68	9.76	92	9.20		2	0.93	11	3.86	13	2.60	
	> 12	33	10.89	108	15.49	141	14.10		1	0.47	5	1.75	6	1.20	
Marital status	Single	100	33.00	330	47.35	430	43.00	Chi^2^ = 26.878; <0.001	10	4.65	56	19.65	66	13.20	Chi^2^ = 33.323; <0.001
	Divorced	20	6.60	35	5.02	55	5.50		5	2.33	7	2.46	12	2.40	
	Married	159	52.48	313	44.91	472	47.20		184	85.58	218	76.49	402	80.40	
	Widowed	24	7.92	19	2.73	43	4.30		16	7.44	4	1.40	20	4.00	
Social support	No	72	23.76	91	13.06	163	16.30	Chi^2^ = 17.744; <0.001	22	10.23	18	6.32	40	8.00	Chi^2^ = 2.554; 0.110
	Yes	231	76.24	606	86.94	837	83.70		193	89.77	267	93.68	460	92.00	

**Table 3:** Economic, psychosocial and health related characteristics of the general sample by self-rated health (N = 1500).

Variables	Categories	DAKAR							TESSEKERE						
		Very poor/Poor		Good/verygood/Excellent		Total		Test	Very poor/Poor		Good/verygood/Excellent		Total		Test
		N	%	N	%	N	%		N	%	N	%	N	%	
Economic well-being	Have difficulty making ends meet	53	17.49	56	8.03	109	10.90	Chi^2^ = 20.318; <0.001	51	23.72	22	7.72	73	14.60	Chi^2^ = 28.761; <0.001
	Live OK but have to be carefull	47	15.51	114	16.36	161	16.10		52	24.19	104	36.49	156	31.20	
	Live OK	160	52.81	399	57.25	559	55.90		91	42.33	121	42.46	212	42.40	
	Live well	43	14.19	128	18.36	171	17.10		21	9.77	38	13.33	59	11.80	
Arterial hypertension	No HTA	211	69.64	543	77.91	754	75.40	Chi^2^ = 7.784; 0.005	129	60.00	214	75.09	343	68.60	Chi^2^ = 12.951; <0.001
	HTA	92	30.36	154	22.09	246	24.60		86	40.00	71	24.91	157	31.40	
Body Mass Index	BMI < 18.5	39	12.87	85	12.20	124	12.40	Chi^2^ = 15.602; 0.001	67	31.16	82	28.77	149	29.80	Chi^2^ = 5.769; 0.123
	18.5 ≤ BMI < 25	156	51.49	426	61.12	582	58.20		105	48.84	165	57.89	270	54.00	
	25 ≤ BMI < 30	63	20.79	134	19.23	197	19.70		36	16.74	30	10.53	66	13.20	
	BMI of ≥ 30	45	14.85	52	7.46	97	9.70		7	3.26	8	2.81	15	3.00	
Glycemia	< 126 md/dL	278	91.75	679	97.42	957	95.70	Chi^2^ = 16.489; <0.001	205	95.35	274	96.14	479	95.80	Chi^2^ = 0.191; 0.662
	≥ 126 mg/dL	25	8.25	18	2.58	43	4.30		10	4.65	11	3.86	21	4.20	
Stress		18,37 ± 5,786		15,74 ± 5,455				t = 6.856; <0.001	18.55± 5.820		15.75 ± 5.793				t = 5,345; <0.001
Total		303	100	697	100	1000	100		215	100	285	100	500	100	

### Multivariate analyses by territory

Table 4 presents the results of logistic regression for poor self-rated health. In Dakar, all things being equal, only gender, age, social support, stress and diabetes remain significant predictors of self-rated health. Women, people aged 50 years and over, those without social support, experiencing stress and diabetics had poorer self-rated health respectively than men, people aged 20 to 49, not stressed, those with social support and non-diabetic individuals.

**Table 4:** Adjusted odds ratio (OR) for poor self-rated health in Dakar and Tessekere (N = 1500).

Variables	Categories	DAKAR (N = 1000)			TESSEKERE (N = 500)		
		p	Odds Ratios	CI for OR (95%)	p	Odds Ratios	CI for OR (95%)
Sex (Men)	Women	.001**	1.728	1.249–2.392	.029*	1.644	1.051–2.571
Age bracket (≥ 50)	20–29	.011*	.497	.289-.854	< .0001**	.172	.089-.336
	30–39	.001**	.436	.262-.725	.001*	.336	.175-.647
	40–49	.306	.764	.457–1.278	.034*	.467	.231-.944
Education level (> 12 years)	0 year	.967	.988	.569–1.718	.760	1.449	.135–15.607
	1–5 years	.679	1.110	.678–1.815	.806	.738	.065–8.359
	6–9 years	.897	1.036	.608–1.765	.949	1.090	.077–15.402
	9–12 years	.460	.783	.409–1.498	.595	.460	.026–8.032
Marital status (Single)	Widowed	.283	1.579	.686–3.634	.259	2.423	.521–11.282
	Married	.660	1.091	.739–1.611	.378	1.471	.623–3.469
	Divorced	.683	1.149	.590–2.237	.999	1.001	.197–5.082
Economic well-being (Live well)	Have difficulty making ends meet	.158	1.516	.851–2.701	.018*	2.896	1.198–7.006
	Live ok but have to be careful	.299	.752	.440–1.287	.155	.585	.279–1.225
	Live ok	.536	.876	.575–1.334	.901	1.045	.520–2.101
Social support (No)	Yes	.007*	.599	.412-.869	.713	.862	.392–1.897
Stress	Continuous	< .001**	1.078	1.049–1.108	< .0001**	1.089	1.047–1.131
Hypertension (No)	Yes	.571	1.110	.774–1.593	.410	1.223	.758–1.974
Body Mass Index (≥ 30 kg/m^2^)	BMI < 18.5	.581	1.198	.631–2.272	.253	2.071	.595–7.211
	18.5 ≤ BMI < 25	.281	.756	.454–1.257	.844	1.130	.336–3.798
	25 ≤ BMI < 30	.363	.775	.448–1.342	.166	2.485	.686–9.007
Glycemia (< 126 mg/dL)	≥ 126 mg/dL	.006*	2.569	1.305–5.055	.826	1.123	.401–3.142

*p<0.05

**p<0.005

In Tessekere, all things being equal, only gender, age, stress and material well-being remain significant predictors of self-rated health. Women, people aged 50 years and over, those who report they have trouble making ends meet and people experiencing stress had poorer self-rated health than their counterparts. It should be noted that age is a stronger predictor of self-rated health in Tessekere than in Dakar, while diabetes in Dakar and economic well-being in Tessekere are the next strongest predictors of this same variable.

Last, education level, marital status, HTN and BMI do not appear associated with self-rated health, either in the urban or rural area.

## Discussion

Overall, our results showed that, in accordance with the majority of studies on the subject, SRH in Senegalese urban area (Dakar) is better than SRH in a rural area (Tessekere). The socioeconomic, demographic and biological differences observed between the two populations might partly explain this tendency: the Dakar population is in fact better educated, younger (and thus more often single) and suffers less from hypertension than the population of Tessekere municipality. Furthermore, Dakar inhabitants enjoy greater economic well-being than Tessekere inhabitants. According to the literature and the data given in Table 2, all these factors could explain the better perceived health in Dakar. But as many articles on self-rated health indicate, “Different cultures provide different frameworks for health evaluations” (i.e. [6]) and it seems clear that Dakar culture is very different from the culture in the Senegalese Ferlo. The young, urban, educated population that lives well in Senegal’s capital is indeed poles apart from the rural, geographically isolated and economically deprived population of Tessekere. Moreover, the determinants of self-rated health partly differ between these two populations, placing a considerable limit on the possibility of make a direct comparison between the proportions of people who report they are in good or poor health.

### Common determinants of self-rated health

Age and gender play a fundamental role in self-rated health as much in Dakar as in Tessekere: older adults and women, as for most populations [22, 27, 49, 50], rate their health more negatively than others. As other authors have suggested, it might be that women are less “stoic” than men when faced with health problems and pay more attention to minor problems, such as headaches, in their subjective health evaluation [51]. This gender paradox—that women have worse self-rated health but lower mortality rates—has been fully studied in western countries [52, 53] and authors found multiple causes, including fundamental biological differences between the sexes, such as genetic factors, immune system response, hormones, and disease patterns, as well as social and behavioral differences.

Stress, a psychosocial variable rarely mentioned in studies on self-rated health, also plays an undeniable role in the populations studied here. In both environments studied, stressed individuals were more likely to rate their health negatively than people who did not experience stress. As Darviri et al. [49] have pointed out in one of the rare studies conducted on this topic, stress is associated with SRH and mortality. To explain this relationship, the authors in particular note, “Excessive stress responses have been linked with maladaptive coping behaviours (such as unhealthy diet, smoking etc.) and various non-infectious diseases of modern societies such as diabetes mellitus, metabolic syndrome, cardiovascular diseases, mediated by the emergence of altered psychoendocrinoimmune responses (e.g., elevated cortisol and catecholamines, pro-inflammatory cytokines, etc.).” It would thus seem that this relationship is not specific to developed countries, but that it also affects populations in low-income countries, even in the most isolated areas such as Tessekere municipality.

Finally, it is worth noting that education, overweight, marital status and hypertension, introduced in binary logistic regression are not associated with SRH neither in Dakar nor in Tessekere. According to the literature, in Ghana [41] or Burkina Faso [31], education level is not associated to SRH, whereas in South Africa, persons with secondary and tertiary education were more likely to report good health [22]. In Senegal, the epidemiological transition is already underway. During the first stage of this transition, very bad and bad health are reported by educated people, whereas at the end of the transition, low educational level is often a predictor of poor self-rated health. It is possible that, Senegal being in the middle of this transition, a balance can exist between low and high educated people, explaining the absence of significance in our analysis. Furthermore, hypertension is not associated with SRH in our study, a result that may be linked to the fact that we used a direct measure of hypertension and not a declared one. It has indeed been demonstrated that hypertensive status based on measured blood pressure did not predict SRH independently of labelling, whereas a consistent and substantial association exists between hypertension labelling and lower SRH [54]. In addition, the lack of association between SRH and marital status may be explained by not taking polygamy into account. Indeed, Macia et al. [55] suggested that polygamy in Senegal is sometimes linked to tensions within couples and households that might lessen the positive impact of monogamous unions in the analysis. Lastly, in our study as in others (i.e. [31, 49]), BMI is not associated to SRH, which may be explained by cultural values associated with overweight or obesity in Senegal: Cohen et al. [56] have shown that plumpness in Senegalese women is more perceived as a symbol of peace and wealth in the household than a risk of disease. Consequently, Senegalese people might not associate overweight or obesity to a poor health.

### The specific determinants of self-rated health

As our results show, being diabetic, in other words having a fasting capillary blood sugar level of ≥126 mg/dL, increases the likelihood of negative self-rated health among Dakar inhabitants, but not among Tessekere inhabitants. This biological variable, which is a true measure of the illness and not a mere declaration of it (as is often the case in other studies), drastically increases (i.e., by 2.5 times) the likelihood of self-reported poor health. Several factors could explain this association between diabetes and self-rated health in Dakar. First of all, more than in other parts of the country, people in Dakar are more aware of the risks of diabetes and are thus certainly more able to identify symptoms associated with the disease, which is now widespread in Senegal’s capital [57]. These two factors certainly explain the relationship between self-rated health and diabetes observed in Dakar.

Social support appears to be associated with self-rated health in Senegal’s capital, whereas this is not the case in Tessekere municipality. Social support has been described as being associated with SRH in several populations [22,40,58,59]. As Jyhla [6] points out, social support is one of the variables that are likely to show an association with self-rated health because it shapes the frameworks of evaluations of self-rated health. In a collectivist society such as Senegal, social relations are of prime importance, and are for instance described as one of the fundamental dimensions of the quality of life [60]. It is hence surprising not to find this influence in Tessekere; but as the results indicate, nearly the entire rural population studied (92%) claims to enjoy social support (Table 1), and this percentage is lower in the urban context of Dakar where ties are dissolved. This certainly explains why social support is an important factor in self-rated health in Dakar, where being able to count on a family member or friend if a problem arises is essential, whereas it is not relevant in Tessekere, a rural setting where social ties remain strong.

Lastly, the results of this study also show that economic well-being plays an important role in self-rated health among Tessekere inhabitants: people who have trouble making ends meet are more than twice as likely to rate their health negatively than others. Saying one has difficulty getting by in Tessekere municipality is tantamount to saying one has financial difficulties in a context where life is subject to many constraints: geographically isolated from the rest of the country (health centers, roads and stores located more than 5 km from camps, without motorized vehicles), Tessekere inhabitants are one of the country’s poorest populations and their lifestyle revolves around livestock breeding and transhumance. Saying one has difficulty making ends meet in a context where difficulty is already the norm amounts to situating oneself among the most destitute people in the country, in a situation of extreme poverty, which probably explains the relationship observed between economic well-being and SRH in Tessekere. The fact that this variable is not associated with SRH in Dakar can be explained by the fact that economic well-being, and particularly the idea of “not able to make ends meet,” holds very different meaning in a privileged urban context such as Dakar where for instance it is far less frequent to have experienced a lack of food at any time during the year than in the rural environment [61].

## Conclusion

The purpose of this exploratory study was to compare SRH in Dakar and in Tessekere and determine the main predictors of self-rated health in both environments. Overall, our results showed that self-rated health is better in Dakar than in Tessekere. As the literature often indicates, people living in rural areas exhibit a less favorable health status than those in urban areas, and consequently, self-rated health is also worse. This is also true in Senegal, and is explained at once by demographic effects (a younger urban population) and socio-economic characteristics: the urban population of Dakar enjoys greater economic well-being, and has far more basic facilities (electricity, running water) and social services (hospitals, health centers, schools, paved roads). But as many authors have pointed out, the definition and process of formulating an answer to the question of self-rated health differ according to individuals’ physical and cultural environments, thus making direct comparison difficult between proportions of people stating they are in good or poor health in Dakar and in Tessekere.

The analyses carried out in these two environments show that despite the existence of common determinants (age, gender, stress), the determinants for formulating an answer to the question of self-rated health differ. While diabetes and social support play a major role in self-rated health in Dakar, this is not the case in the rural environment. In Tessekere, it is instead economic well-being that has a significant influence on self-rated health. People’s social and cultural environments (viewed in particular through access to health care, economic resources, representations of illness, the importance of social relations and also stress) thus play a fundamental role in the process of rating one’s health and, in the short and long term, in the mortality rate. Finally, it is important to note that Tessekere is a very small zone, whereas rural area is very diverse in Senegal. Consequently, results from our study can’t be regarded as representative of the entire rural area in Senegal.

In the future, it will be necessary to verify that self-rated health is a predictor of mortality in West Africa. Similar research has been carried out in nearly all countries of the world, but not south of the Sahara (with the exception of South Africa). Such data is essential to determine the invariant character of this relationship. Research on self-rated health is only just starting in sub-Saharan Africa, but the heuristic potential of this field of study appears to clarify the still unresolved issues in international literature.

## Supporting information

S1 File Questionnaire in English. (DOC)

S2 File Questionnaire original language. (DOC)

S3 File Database. (XLSX)

## References

[1] World Health Organization. Constitution of the World Health Organization—Basic Documents (4^th^ ed). Geneva:WHO;2006.

[2] Diener E, Chan MY. Happy people live longer: subjective well-being contributes to health and longevity. Appl Psychol Health Well Being. 2011;3:1–43

[3] Nyamwaya D. Health promotion in Africa: strategies, players, challenges and prospects. Health Promot Int. 2003;18:85–7. 12746379 10.1093/heapro/18.2.85

[4] Idler EL, Benyamini Y. Self-rated health and mortality: a review of twenty-seven community studies. J Health Soc Behav. 1997;38:21–37. 9097506

[5] Idler EL, Kasl SV. Self-ratings of health: do they also predict change in functional ability? J Gerontol B Psychol Sci Soc Sci. 1995;50:S344–53. 7583813 10.1093/geronb/50b.6.s344

[6] Jylhä M. What is self-rated health and why does it predict mortality? Towards a unified conceptual model. Soc Sci Med. 2009;69:307–16. doi: 10.1016/j.socscimed.2009.05.013 19520474 10.1016/j.socscimed.2009.05.013

[7] Mauss M. Rapports réels et pratiques de la psychologie et de la sociologie In: Mauss M, editor. Sociologie et anthropologie. Paris:Presses Universitaires de France; 1924 p. 280–310.

[8] Baron-Epel O, Kaplan G, Haviv-Messika A, Tarabeia J, Green MS, Kaluski DN. Self-reported health as a cultural health determinant in Arab and Jewish Israelis MABAT—National Health and Nutrition Survey 1999–2001. Soc Sci Med. 2015;61:1256–66. 10.1016/j.socscimed.2005.01.022 15970235

[9] Desquelles AF, Egidi V, Salvatore MA. Why do Italian people rate their health worse than the French people do? An exploration of cross-country differences of self-rated health. Soc Sci Med. 2009;68:1124–28. doi: 10.1016/j.socscimed.2008.12.037 19157664 10.1016/j.socscimed.2008.12.037

[10] Ringen S. Wellbeing, measurement, and preferences. Acta Sociol. 1995;38:3–15.

[11] Salmon P, Dowrick CF, Ring A, Humphris GM. Voiced but unheard agendas: qualitative analysis of the psychosocial cues that patients with unexplained symptoms present to general practitioners. Br J Gen Pract. 2004;54:171–76. 15006121 PMC1314826

[12] Patrick DL, Erickson P. Health status and health policy: quality of life in health care evaluation and resource allocation. New York: Oxford University Press;1993.

[13] Chandola T, Crispin J. Validating self-rated health in different ethnic groups. Ethn Health. 2000;5:151–9. doi: 10.1080/713667451 10984833 10.1080/713667451

[14] Frankenberg E, Jones NR. Self-rated health and mortality: does the relationship extend to a low income setting? J Health Soc Behav. 2004;45:441–52. doi: 10.1177/002214650404500406 15869115 10.1177/002214650404500406

[15] Rahman MO, Barsky AJ. Self-reported health among older Bangladeshis: how good a health indicator is it? Gerontologist. 2003;43:856–63. 14704385 10.1093/geront/43.6.856

[16] Addai I, Adjei J. Predictors of self-appraised health status in sub-Saharan Africa: the case of Ghana. Appl Res Qual Lif. 2014;9:233–53.

[17] Chin B. Income, health, and well-being in rural Malawi. Demogr Res. 2010;23:997–1030. doi: 10.4054/DemRes.2010.23.35 21359133 10.4054/DemRes.2010.23.35 PMC3045086

[18] Debpuur C, Welaga P, Wak G, Hodgson A. Self-reported health and functional limitations among older people in the Kassena-Nankana District, Ghana. Glob Health Action. 2010;3:54–63. 10.3402/gha.v3i0.2151 PMC2957305 20963186

[19] Obare F. Self-rated health status and morbidity experiences of teenagers in Nairobi’s low income settings. Afr Popul Stud. 2007;22:57–74.

[20] Kuate-Defo B. Facteurs associés à la santé perçue et à la capacité fonctionnelle des personnes âgées dans la préfecture de Bandjoun au Cameroun. Cah Qué Démogr. 2005;34:1–46.

[21] Gilbert L, Soskolne V. Self-assessed health—A case study of social differentials in Soweto, South Africa. Health Place. 2003;9:193–205. 12810327 10.1016/s1353-8292(02)00039-4

[22] Chola L, Alaba O. Association of neighbourhood and individual social capital, neighbourhood economic deprivation and self-rated health in South Africa—A multi-level analysis. PloS One. 2013; doi: 10.1371/journal.pone.0071085 23976923 10.1371/journal.pone.0071085 PMC3743525

[23] Ramlagan S, Pelzer K, Phaswana-Mafuya N. Social capital and health among older adults in South Africa. BMC Geriatr. 2013;13:100 doi: 10.1186/1471-2318-13-100 24073666 10.1186/1471-2318-13-100 PMC3851859

[24] Cramm JM, Nieboer AP. The influence of social capital and socio-economic conditions on self-rated health among residents of an economically and health-deprived South African township. Int J Equity Health. 2011;10: doi: 10.1186/1475-9276-10-51 22085826 10.1186/1475-9276-10-51 PMC3252245

[25] Coumé M, Touré K, Faye A, Sall S, Pouye A, Diop TM. Facteurs prédictifs de mauvaise santé perçue chez les femmes âgées sénégalaises. Les Cahiers de l’Année Gérontologique. 2014;6:90–5.

[26] Tomas JM, Gutiérrez M, Sancho P, Galiana L. Predicting perceived health in Angolan elderly: the moderator effect of being oldest old. Arch Gerontol Geriatr. 2012;55:605–10. doi: 10.1016/j.archger.2012.06.010 22770711 10.1016/j.archger.2012.06.010

[27] Onadja Y, Atchessi N, Soura BA, Rossier C, Zunzunegui MV. Gender differences in cognitive impairment and mobility disability in old age: a cross-sectional study in Ouagadougou, Burkina Faso. Arch Gerontol Geriatr. 2013;57:311–8. doi: 10.1016/j.archger.2013.06.007 23827740 10.1016/j.archger.2013.06.007

[28] Macia E, Duboz P, Montepare JM, Gueye L. Age identity, self-rated health and life satisfaction among older adults in Dakar, Senegal. Eur J Ageing. 2012;9:243–253. doi: 10.1007/s10433-012-0227-7 28804424 10.1007/s10433-012-0227-7 PMC5547411

[29] Diouf AB, Touré K, Coumé M, Diop AG, Ndiaye MM. Predictors of poor self-rated health in a Senegalese male elderly population utilizing the social and health center of Ipres, Senegal. J Neurol Sci. 2014;333:e663.

[30] Ardington C, Gasealahwe B. Mortality in South Africa: Socio-economic profile and association with self-reported health. Dev South Afr. 2014;31(1):127–145. doi: 10.1080/0376835X.2013.853611 24436509 10.1080/0376835X.2013.853611 PMC3890238

[31] Onadja Y, Bignami S, Rossier C, Zunzunegui MV. The components of self-rated health among adults in Ouagadougou, Burkina Faso. Popul Health Met. 2013;11:15. 10.1186/1478-7954-11-15 PMC3750468 23926951

[32] Australian Institute of Health and Welfare. Rural, regional and remote health: A study on mortality. 2007 Rural health series 8.

[33] Pampalon R, Martinez J, Hamel D. Does living in rural areas make a difference for health in Quebec? Health Place. 2006;12(4):421–435. doi: 10.1016/j.healthplace.2005.04.002 15955720 10.1016/j.healthplace.2005.04.002

[34] Canadian Institute for Health Information. How Healthy are Rural Canadians? An Assessment of Their Health Status and Health Determinants. Ottawa: Canadian Institute for Health Information;2006

[35] Eberhardt MS, Ingram DD, Makuc DM. Urban and Rural Health Chartbook Health, United States. Hyattsville: National Center for Health Statistics;2001

[36] Ministère de la Santé et de la Prévention Médicale. Enquête démographique et de santé. Dakar-Maryland:Centre de Recherche pour le Développement Humain-ORC Macro; 2005

[37] Tabutin D, Schoumaker B. La démographie de l’Afrique au sud du Sahara des années 1950 aux années 2000. Synthèse des changements et bilan statistique. Population. 2004;59(3–4):521–622.

[38] Arokiasamy P, Uttamacharya U, Jain K, Biritwum RB, Yawson AE, Wu F, et al The impact of multimorbidity on adult physical and mental health in low-and middle-income countries: what does the study on global ageing and adult health (SAGE) reveal? BMC medicine. 2015;13(1):178. 26239481 10.1186/s12916-015-0402-8 PMC4524360

[39] Cantrelle P. Démographie et Santé. Journées Médicales de Dakar, janvier 1982, http://horizon.documentation.ird.fr/exl-doc/pleins_textes/pleins_textes_5/b_fdi_16-17/21269.pdf

[40] Lee JA, Park JH, Kim M. Social and physical environments and self-rated health in urban and rural communities in Korea. Int J Environ Res Public Health. 2015;12(11):14329–14341. doi: 10.3390/ijerph121114329 26569279 10.3390/ijerph121114329 PMC4661651

[41] Udofia EA, Yawson AE, Aduful KA, Bwambale FM. Residential characteristics as correlates of occupants’ health in the greater Accra region, Ghana. BMC public health. 2014;14(1):244. 24612884 10.1186/1471-2458-14-244 PMC3995710

[42] Lankila T, Näyhä S, Rautio A, Nordström T, Koiranen M, Taanila A, et al Self-reported health in urban-rural continuum: a grid-based analysis of Northern Finland Birth Cohort 1966. Int J Public Health. 2012;57(3):525–533. doi: 10.1007/s00038-011-0286-0 21853282 10.1007/s00038-011-0286-0

[43] United Nations. Human Development Report 2015. 2015. http://hdr.undp.org/en/content/human-development-report-2015

[44] Senegal's national statistical office. General census of Populations, Dwellings, Agriculture and Breeding Report (RGPHAE, 2013). Dakar: UNFPA/USAID, 2015

[45] Ndiaye S, Ayad M. Enquête démographique et de santé–Sénégal 2005. Centre de Recherche pour le Développement Humain (Sénégal) et ORC Macro, Calverton (USA). 2006. http://www.measuredhs.com/pubs/pdf/FR177/FR177.pdf

[46] Cohen S, Kamarck T, Mermelstein R. A global measure of perceived stress. J Health Soc Behav. 1983;385–396. 6668417

[47] Kumar S, Calvo R, Avendano M, Sivaramakrishnan K, Berkman LF. Social support, volunteering and health around the world: Cross-national evidence from 139 countries. Soc Sci Med. 2012;74(5):696–706. doi: 10.1016/j.socscimed.2011.11.017 22305947 10.1016/j.socscimed.2011.11.017

[48] Razafindrakoto M, Roubaud F. Les déterminants du bien-être individuel en Afrique francophone: le poids des institutions. Afr Contemp. 2006;220:191–223.

[49] Darviri C, Fouka G, Gnardellis C, Artemiadis AK, Tigani X, Alexopoulos EC. Determinants of self-rated health in a representative sample of a rural population: a cross-sectional study in Greece. Int J Environ Res Public Health. 2012;9(3):943–954. doi: 10.3390/ijerph9030943 22690175 10.3390/ijerph9030943 PMC3367289

[50] Hosseinpoor AR, Williams JS, Amin A, De Carvalho IA, Beard J, Boerma T, et al Social determinants of self-reported health in women and men: understanding the role of gender in population health. PLoS One. 2012;7(4):e34799 doi: 10.1371/journal.pone.0034799 22514667 10.1371/journal.pone.0034799 PMC3326052

[51] Spiers N, Jagger C, Clarke M, Arthur A. Are gender differences in the relationship between self-rated health and mortality enduring? Results from three birth cohorts in Melton Mowbray, United Kingdom. Gerontologist. 2003;43:406–11. 12810905 10.1093/geront/43.3.406

[52] Oksuzyan A, Juel K, Vaupel JW, Christensen K. Men: good health and high mortality. Sex differences in health and aging. Aging Clin Exp Res. 2008; 20(2): 91–102. 18431075 10.1007/bf03324754 PMC3629373

[53] Crimmins EM, Kim JK, Sole-Auro A. Gender differences in health: results from SHARE, ELSA and HRS. Eur J *Public Health*. 2011; 21(1): 81–91. doi: 10.1093/eurpub/ckq022 20237171 10.1093/eurpub/ckq022 PMC3023013

[54] Barger SD, Muldoon MF. Hypertension labelling was associated with poorer self-rated health in the Third US National Health and Nutrition Examination Survey. J Hum Hypertens. 2006;20(2),117–23. doi: 10.1038/sj.jhh.1001950 16267563 10.1038/sj.jhh.1001950

[55] Macia E, Duboz P, Montepare JM, Gueye L. Exploring life satisfaction among older adults in dakar. J Cross Cult Gerontol. 2015;30:377–391. doi: 10.1007/s10823-015-9275-8 26481797 10.1007/s10823-015-9275-8

[56] Cohen E. La construction sociale du corps chez les Sénégalais, une clé pour appréhender leur rapport à la corpulence dans le contexte de la transition des modes de vie. Ph.D. Dissertation in Biological Anthropology, Aix-Marseille University. 2012.

[57] Duboz P, Chapuis-Lucciani N, Boëtsch G, Gueye L. Prevalence of diabetes and associated risk factors in a Senegalese urban (Dakar) population. Diabetes Metab. 2012;38(4):332–336. doi: 10.1016/j.diabet.2012.02.011 22521041 10.1016/j.diabet.2012.02.011

[58] Poortinga W. Social relations or social capital? Individual and community health effects of bonding social capital. Soc Sci Med. 2006;63:255–270. doi: 10.1016/j.socscimed.2005.11.039 16427171 10.1016/j.socscimed.2005.11.039

[59] Ellaway A, Macintyre S. Social capital and self-rated health: support for a contextual mechanism. Am J Public Health. 2000;90:988. 10.2105/ajph.90.6.988a PMC1446269 10877731

[60] Macia E, Duboz P, Gueye L. Les dimensions de la qualité de vie subjective à Dakar. Sci Soc Santé. 2010;28:75–84.

[61] Agence Nationale de la Statistique et de la Démographie. Pauvreté et conditions de vie des ménages. Dakar:Agence Nationale de la Statistique et de la Démographie; 2011. http://www.ansd.sn/ressources/publications/PAUVRETE%20ET%20CONDITION%20DE%20VIE%20DES%20MENAGES-DEF-VRC-VF.pdf

