# Supplementary material for: Self-rated health in Senegal: A comparison between urban and rural areas
Source: PLoS One. 2017 Sep 8;12(9):e0184416. doi: 10.1371/journal.pone.0184416 (PMC5590920; doi:10.1371/journal.pone.0184416)
Supplement: S1 File — (DOC) [file pone.0184416.s001.doc]

**QUESTIONNAIRE ECOSAN 2015**

**CIVIL STATUS**

1. ID : ……………… 2. Sex: 3. Age:……………….

3. Home address :…………………………………………………………………………..

**SOCIAL SUPPORT**

If you were in trouble, do you have friends and relatives you can count on to help you whenever you need them, or not?

Yes 🞏

No 🞏

**PERCEIVED STRESS SCALE**

The questions in this scale ask you about your feelings and thoughts during the last month

1- how often have you been upset because of something that happened unexpectedly?

Never

Almost Never

Sometimes

Fairly Often

Very Often

2- how often have you felt that you were unable to control the important things in your life?

Never

Almost Never

Sometimes

Fairly Often

Very Often

3- how often have you felt nervous and “stressed”?

Never

Almost Never

Sometimes

Fairly Often

Very Often

4- how often have you felt confident about your ability to handle your personal problems?

Never

Almost Never

Sometimes

Fairly Often

Very Often

5- how often have you felt that things were going your way?

Never

Almost Never

Sometimes

Fairly Often

Very Often

6- how often have you found that you could not cope with all the things that you had to do?

Never

Almost Never

Sometimes

Fairly Often

Very Often

7- how often have you been able to control irritations in your life?

Never

Almost Never

Sometimes

Fairly Often

Very Often

8- how often have you felt that you were on top of things?

Never

Almost Never

Sometimes

Fairly Often

Very Often

9- how often have you been angered because of things that were outside of your control?

Never

Almost Never

Sometimes

Fairly Often

Very Often

10- how often have you felt difficulties were piling up so high that you could not overcome them?

Never

Almost Never

Sometimes

Fairly Often

Very Often

**HEALTH**

***SELF-PERCEIVED HEALTH***

Overall, would you say that your health is: excellent, very good, good, fair or poor?

Excellent

Very good

Good

Fair

Poor

Pas du tout

Tout à fait

**SOCIO-DEMOGRAPHIC VARIABLES**

Marital status: Married 🞎 Divorced 🞎 Widowed 🞎 Cohabitation 🞎 Single 🞎

Educationnal level

None 🞏

Koranic school 🞏

Primary education 🞏

Lower secondary education 🞏

Upper secondary education 🞏

Academic cycle 🞏

Given your household income, do you feel you:

Live well 🞎

Live okay 🞎

Live okay, but you have to be careful 🞎

Have difficulty making ends meet 🞎

**BIOLOGICAL VARIABLES**

Arterial pressure :

Weight :…………………… kgs

Height :…………………… cms

Glycemia :
