# Supplementary material for: Self-rated health in Senegal: A comparison between urban and rural areas
Source: PLoS One. 2017 Sep 8;12(9):e0184416. doi: 10.1371/journal.pone.0184416 (PMC5590920; doi:10.1371/journal.pone.0184416)
Supplement: S2 File — (DOC) [file pone.0184416.s002.doc]

**QUESTIONNAIRE ECOSAN 2015**

**ETAT CIVIL**

1. Identifiant : ……………… 2. Sexe : 3. Age :………….

3. Lieu de résidence :…………………………………………………………………………..

**SUPPORT SOCIAL**

Si vous aviez des problèmes, auriez-vous des proches et des amis sur lesquels vous pourriez compter pour vous aider à n’importe quel moment ?

Oui 🞏

Non 🞏

**STRESS**

*Je vais désormais vous poser diverses questions concernant vos sensations et pensées pendant le mois qui vient de s'écouler.*

Au cours du dernier mois, combien de fois...

1-… avez-vous été contrarié(e) par un événement inattendu ?

Jamais

Rarement

Parfois

Assez souvent

Très souvent

2-.... vous a-t-il semblé difficile de gérer les choses importantes de votre vie ?

Jamais

Rarement

Parfois

Assez souvent

Très souvent

3-... vous êtes-vous senti(e) nerveux(se) et stressé(e)  ?

Jamais

Rarement

Parfois

Assez souvent

Très souvent

4-... vous êtes-vous senti(e) confiant(e) dans vos capacités à gérer vos problèmes personnels ?

Jamais

Rarement

Parfois

Assez souvent

Très souvent

5-... avez-vous senti que les choses allaient comme vous le vouliez ?

Jamais

Rarement

Parfois

Assez souvent

Très souvent

6-... avez-vous pensé que vous n’arriveriez pas à faire toutes les choses que vous deviez faire ?

Jamais

Rarement

Parfois

Assez souvent

Très souvent

7-... avez-vous été capable de maîtriser votre énervement ?

Jamais

Rarement

Parfois

Assez souvent

Très souvent

8-... avez-vous senti que vous dominiez la situation ?

Jamais

Rarement

Parfois

Assez souvent

Très souvent

9-... vous êtes-vous senti(e) irrité(e) parce que les événements vous échappaient ?

Jamais

Rarement

Parfois

Assez souvent

Très souvent

10-... avez-vous trouvé que les difficultés s'accumulaient à un tel point que vous ne pouviez pas les gérer ?

Jamais

Rarement

Parfois

Assez souvent

Très souvent

**SANTE**

***santé perçue***

Dans l’ensemble, pensez-vous que votre santé est :

Excellente

Très bonne

Bonne

Médiocre

Mauvaise

**VARIABLES SOCIO-DEMOGRAPHIQUES**

***MENAGE***

Etes-vous Marié 🞎 Divorcé 🞎 Veuf(ve) 🞎 En concubinage 🞎 Célibataire 🞎 ?

***Catégorie Socio-Professionnelle et Niveau d’éducation***

Quel est votre niveau d’instruction ?

Absence 🞏

Ecole coranique 🞏

Primaire 🞏

1^er^ cycle secondaire 🞏

2^ème^ cycle secondaire 🞏

Cycle universitaire 🞏

***CONDITIONS ECONOMIQUES ET MATERIELLES***

Etant donné les ressources de votre ménage, vous estimez que vous vivez :

Bien 🞎 Ça va à peu près 🞎 Ça va, mais il faut faire attention 🞎 Difficilement 🞎

**MESURES BIOLOGIQUES**

Tension artérielle :

Poids :…………………… kgs

Taille :…………………… cms

Glycémie :
